# Genome-wide characterization of the UDP-glycosyltransferases (UGT) family and functional analysis of *VcUGT160* involved in dihydrozeatin glycosylation during blueberry fruits development

**DOI:** 10.1186/s12864-025-12267-5

**Published:** 2025-11-14

**Authors:** Yifei Wang, Xinlei Liu, Tong Zhao, Jing Li, Li Chen, Yadong Li, Haiyue Sun

**Affiliations:** 1 Engineering Center of Genetic Breeding and Innovative Utilization of Small Fruits of Jilin Province, College of Horticulture, Jilin Agricultural University, 2888 Xin Cheng Street, Changchun, Changchun, Jilin 130118 PR China; 2 Laboratory of Quality and Safety Risk Assessment for Fruit, Research Institute of Pomology, Chinese Academy of Agricultural Sciences, Xingcheng, Liaoning 125100 PR China

**Keywords:** Blueberry, UGT family, Dihydrozeatin glycosylation, Functional analysis

## Abstract

**Background:**

Blueberry (*Vaccinium spp.*) celebrated for its rich nutritional content and significant health benefits, was referred to as the ‘Queen of Fruits’ and the ‘King of Berries’. The development of blueberry fruits is closely related to plant hormones. Glycosylation mediated by UDP-glycosyltransferases (UGTs) is a key step in plant hormones homeostasis. However, the UGT members has not been reported in blueberry to date.

**Results:**

A total of 361 *VcUGT* genes were identified and classified into 11 groups. Whole-genome and segmental duplications drove *VcUGT* expansion, with structural analysis revealing conservation within subgroups but divergence among them. Group G members were associated with cytokinin glycosylation, particularly dihydrozeatin, which promotes fruit enlargement. RT-qPCR suggested *VcUGTs* involvement in fruit development, while functional validation confirmed VcUGT160 localization to the nucleus and cell membrane, likely mediating dihydrozeatin glycosylation.

**Conclusions:**

The results of this study identified and characterized the UGT gene family in Blueberry. VcUGT160 may function in mediating dihydrozeatin glycosylation. Moreover, these findings enhance our understanding of VcUGTs evolution and function in blueberry.

**Supplementary Information:**

The online version contains supplementary material available at 10.1186/s12864-025-12267-5.

## Background

Glycosylation is a prominent modification that plays crucial roles in plant growth. Glycosyltransferases (GTs, EC 2.4.X.Y), the enzymes that are responsible for glycosylation, distributed in almost all organisms and highly differentiated in functions. As a natural biocatalyst, GTs facilitates glycosylation by catalyzing transfer of sugar molecules from activated donors to specific receptors by the formation of a glycosidic bond [1]. Small molecule glycosylation is mainly catalyzed by uridine diphosphate glucosyltransferases (UDP-Glycosyltransferases, UGTs), belonging to the GT1 family. UGTs protein possess a 44-amino acid PSPG (Plant Secondary Product Glycosyltransferase) motif located near the C-terminal, which is thought to possibly bind specifically to glycosyl donors [2], while the sequences at N-terminus of UGTs are highly diverse and considered to be responsible for recognition of a variety of substrates. In recent years, UGT families have been systematically identified in Arabidopsis (*Arabidopsis thaliana*) [3], maize (*Zea mays*) [4], apple (*Malus domestica*) [5] and citrus (*Citrus sinensis*) [6]. UGT proteins were mainly involved in the biosynthesis of natural plant products such as flavonoids, quinones, steroids and hormones which regulate plant growth and development [7]. Additionally, diverse multi-gene UGT family members function together to modulate complicated biochemical processes in plant cells, which in turn affects numerous biological activities and functions.

Plant hormones are substances produced by plants that regulate metabolism [8], influencing cell division and elongation, tissue and organ differentiation, flowering, fruit development, maturation, aging, seed dormancy, and germination, maintain a dynamic balance by either coordinating or antagonizing, collectively regulating plant growth, development, and differentiation [9]. Glycosylation of plant hormones can reduce or inactivate their levels, serving as a key mechanism for plants to regulate endogenous hormone balance. To date, many glycosyltransferases involved in hormone regulation have been identified, including auxin glycosyltransferases [10–12], abscisic acid glycosyltransferases [8], cytokinin glycosyltransferases [13], salicylic acid glycosyltransferases [14–16], jasmonate glycosyltransferases [17], and brassinosteroid glycosyltransferases [18]. Cytokinin glycosylation is essential for maintaining the dynamic equilibrium of various cytokinin. The two main types of cytokinin glycosylation are *N*-glycosylation and *O*-glycosylation [19, 20]. *O*-glycosylation primarily activates cytokinin by converting them into their active forms through specific *β*-glucosidases [21]. While the glycosyl moieties are transferred by specialized glycosyltransferases from activated glycosyl donors to hydroxyl groups on cytokinin side. This process is particularly important for trans-zeatin and dihydrozeatin, influencing their storage, transport, and the protection against degrading enzymes [22]. Currently, cytokinin glucosyltransferase has been identified from *Phaseolus lunatus*, *Glycine max*, *Oryza sativa*, and *Lycopersicon esculentum*, while cytokinin *O*-xylosyltransferase has primarily been identified *P. vulgaris* [23, 24]. Recent studies have identified UGT85A1(a member of the UGT family) as a trans-zeatin-specific *O*-glucosyltransferase in maize. In Arabidopsis *ugt85a1* mutants, chlorophyll degradation is reduced, anthocyanin accumulation increases, and genes related to senescence and stress responses are activated. In contrast, *UGT85A1* overexpression lines accelerate cytokinin inactivation and promotes root elongation [13]. These findings indicate that members of the UGT85 (A UGT subfamily named according to the HUGO Gene Nomenclature Committee guidelines.) family play a crucial role in regulating organ development through the glycosylation of cytokinin.

Blueberry (*Vaccinium spp.*), a woody plant of Ericaceae [25], known for its fruits, delicate texture and balanced sweet-tart flavor, rich in nutrients, contains high amounts of antioxidants, anthocyanins, and various nutrients that have significant health benefits for the human body, making them one of the most popular health-promoting fruits of the 21 st century [26]. The blueberry fruit is a pseudocarp that develops from an inferior ovary, and its ripening process was regulated by plant hormones such as abscisic acid (ABA), ethylene (ETH), auxin (IAA), and cytokinin (CTK) [27]. So far, research on glycosylation in blueberries remains limited, and the glycosyltransferase family in this species has not yet been characterized. Moreover, studies specifically exploring the role of glycosylation in cytokinin regulation are particularly scarce. Therefore, it is significant and meaningful to identify and analyze the UGT gene family in blueberry.

In this study, the genome of blueberry was used to identify the VcUGT family and obtain potential *VcUGT* genes that might be related to plant hormones glycosylation. A total of 361 *VcUGT* candidate genes were identified and their structure and evolutionary relationship were analyzed. Twelve *UGT* candidate genes were identified with plant hormones glycosylation in group E via expression pattern. Furthermore, subcellular localization and VIGS (virus-induced gene silencing) confirmed the functional location of VcUGT160 and its relationship with the glycosylation of dihydrozeatin.

## Results

### Identification and phylogenetic analysis of UGT family members

The publication of the highbush blueberry variety *‘Draper’* genome greatly facilitated identifying blueberry gene families. To identify blueberry *UGT* family genes, the HMMER3.0 tools were used to Identify gene members with the conserved domain of the PSPG-box (PF00201) in ‘*Draper*’ genome database. After determining the presence of the PSPG domain in all the amino sequences, 361 predicted blueberry VcUGT members were identified (Table S1).

**Fig. 1:**
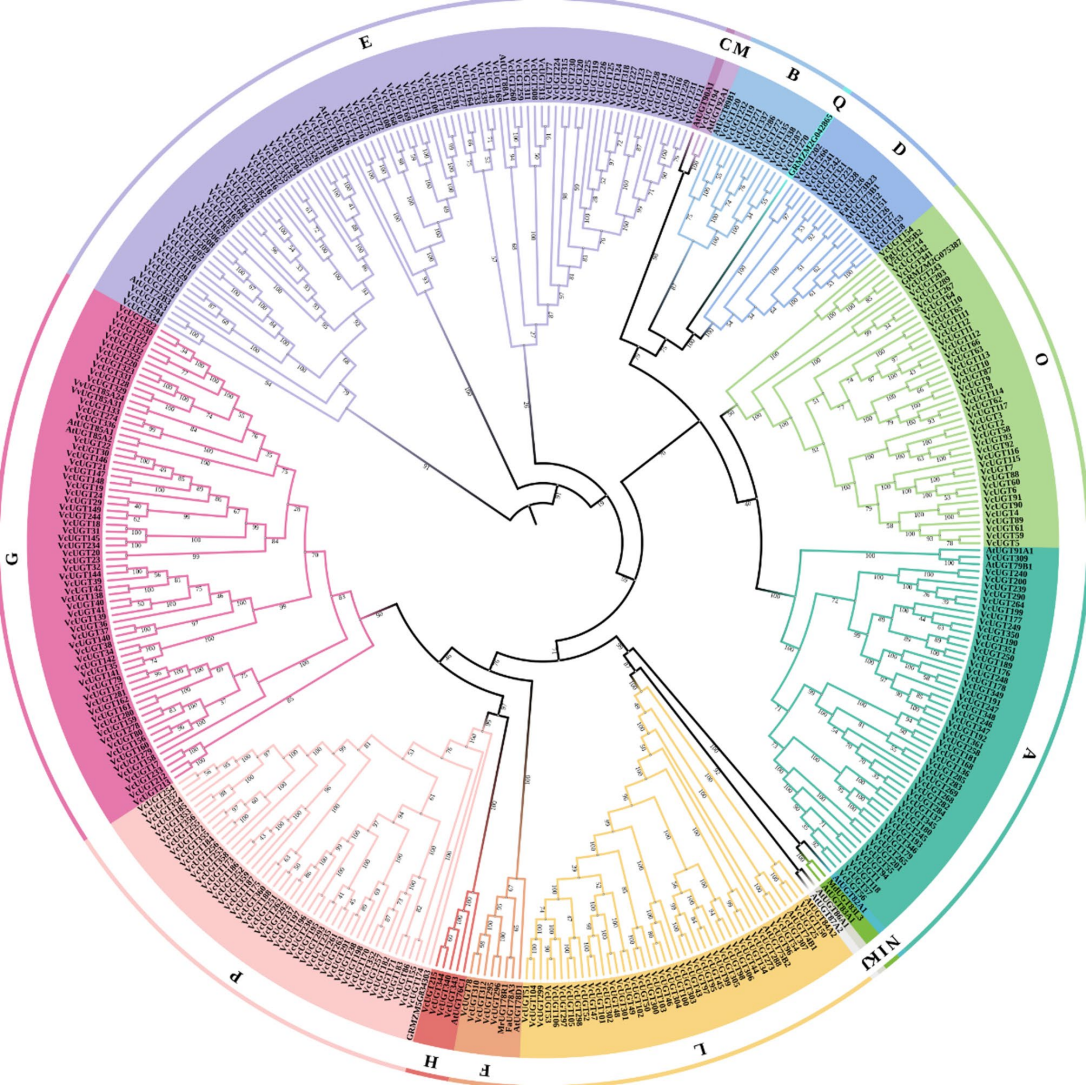
Phylogenetic analysis of blueberry, Arabidopsis, maize and several fruit plants of UGT family. A total of 361 blueberry UGT proteins, 20 Arabidopsis UGT proteins, 3 maize UGT proteins and 7 UGT proteins from fruit plants UGT proteins were included in the phylogenetic tree constructed using a Maximum likelihood method. Different groups of UGT family were marked with colors and letters

A phylogenetic tree of UGT family proteins was constructed by aligning the full-length amino acid sequences of blueberry UGTs with 20 UGTs from Arabidopsis and 10 from other plants (Fig. 1). Based on the plant classification model, blueberry VcUGT family members were divided into 11 groups. The VcUGT count varied drastically across groups, with group E and group G leading the pack with 84 and 64 members, respectively, while group M had a solitary representative. Interestingly, groups C, N, I, J, and K were absent in the blueberry VcUGT family, suggesting evolutionary deletions and potential functional differentiation in different plant UGT family.

### Conserved motif analysis of VcUGT family members

**Fig. 2:**
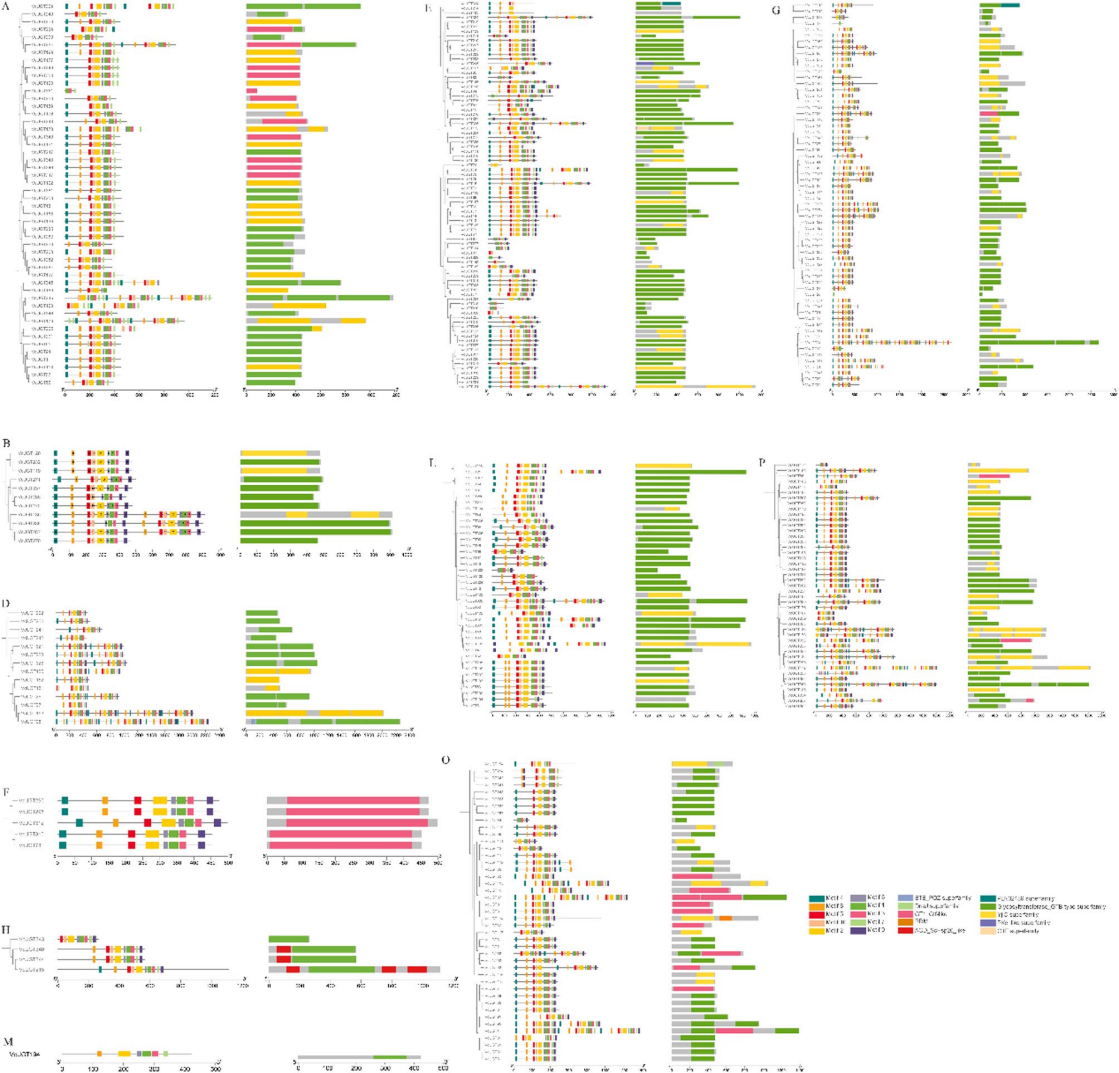
Distribution of 10 signature motifs of obtained from blueberry UGTs proteins using MEME analysis. Different motifs were represented by different colors. Typical structural domains from UGT protein were also showed on the right side

Conserved motifs in VcUGT proteins were identified using the MEME motif search tools. In total, 10 conserved motifs were identified and annotated using SMART protein analysis software. The results indicated a highly similar distribution of UGT members (Fig. 2). Motif 1, the typical PSPG-box, was present in almost all VcUGT members. Members with the PSPG-box exhibited strong conservation in the N-terminal region, while showing considerable variability in the C-terminal region, similar to other plants. This suggests high conservation among family members across different plant species. Additionally, typical structural domains such as GTB, YjiC, and GT1 were found in blueberry UGT members, although most members were annotated with only one domain. This varying distribution of structure may explain functional differences among blueberry UGT family members.

### Gene structural analysis of *VcUGT* family genes

**Fig. 3:**
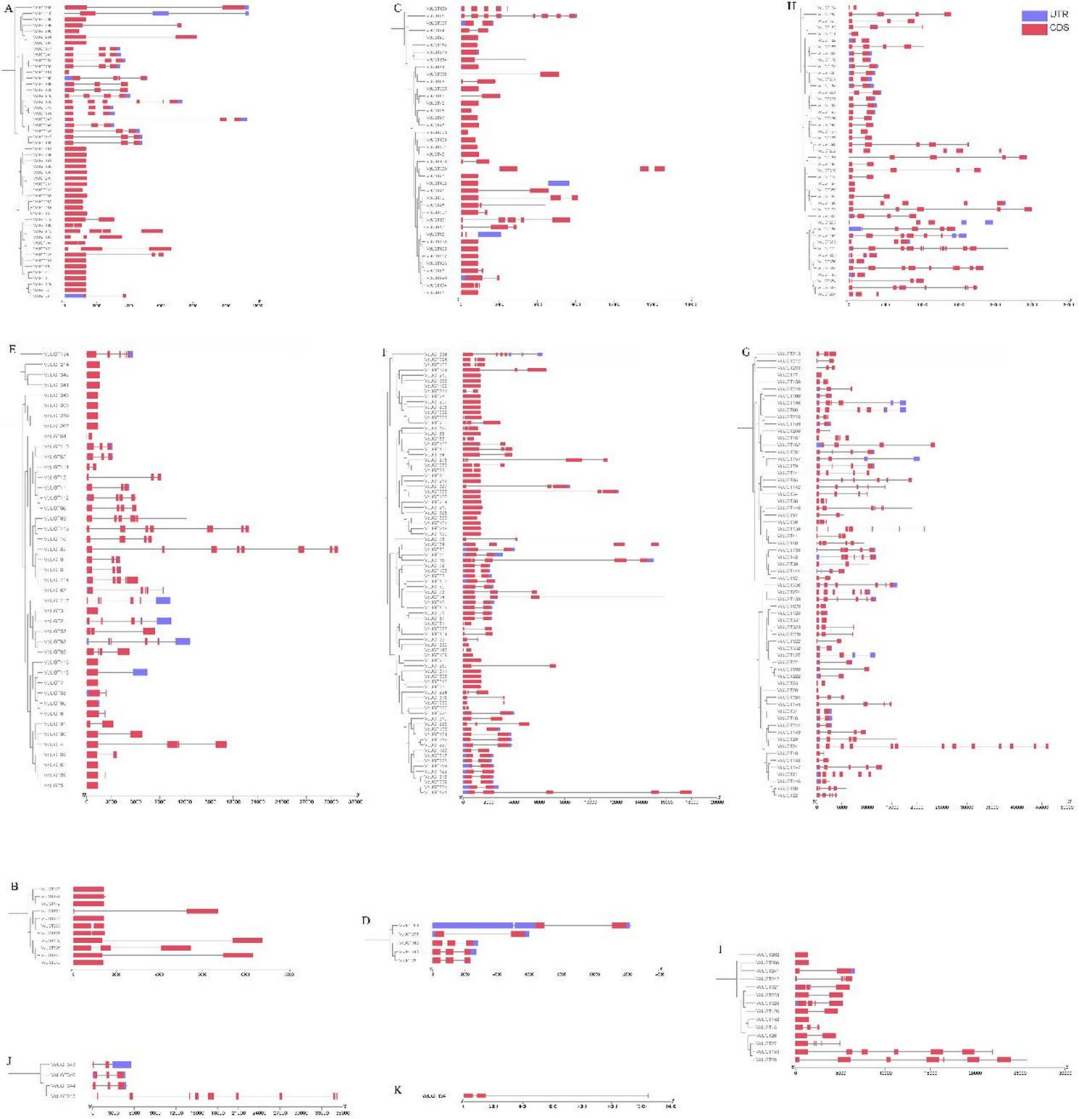
The organization of exon-intron structures of VcUGTs genes. The lines indicate introns, the red boxes indicate exons and the purple box indicate UTRs

To further explore the structural differences and conservative relationship of *VcUGT* genes in different groups, the intron and exon patterns were determined by comparing the full-length CDS and DNA sequences of candidate *VcUGT*s using the GSDS web server. The results showed that the number of intron regions in *VcUGTs* ranged from 1 to 14, and members within the same group exhibited similar intron/exon structural features, including exon numbers and length (Fig. 3). Groups F, G, H, M, and P predominantly contain *UGT* gene members with three or more introns, whereas groups A, B, D, E, L, and O have fewer introns, possibly due to shorter sequences and the loss of intron-rich GTB domains during evolution. These findings suggest significant structural differences among members of the blueberry *VcUGTs* gene across groups, while members of closely related groups share similar structures but may differ in length.

### Analysis of *cis*-regulatory elements in the promoters of *VcUGT* family genes

**Fig. 4:**
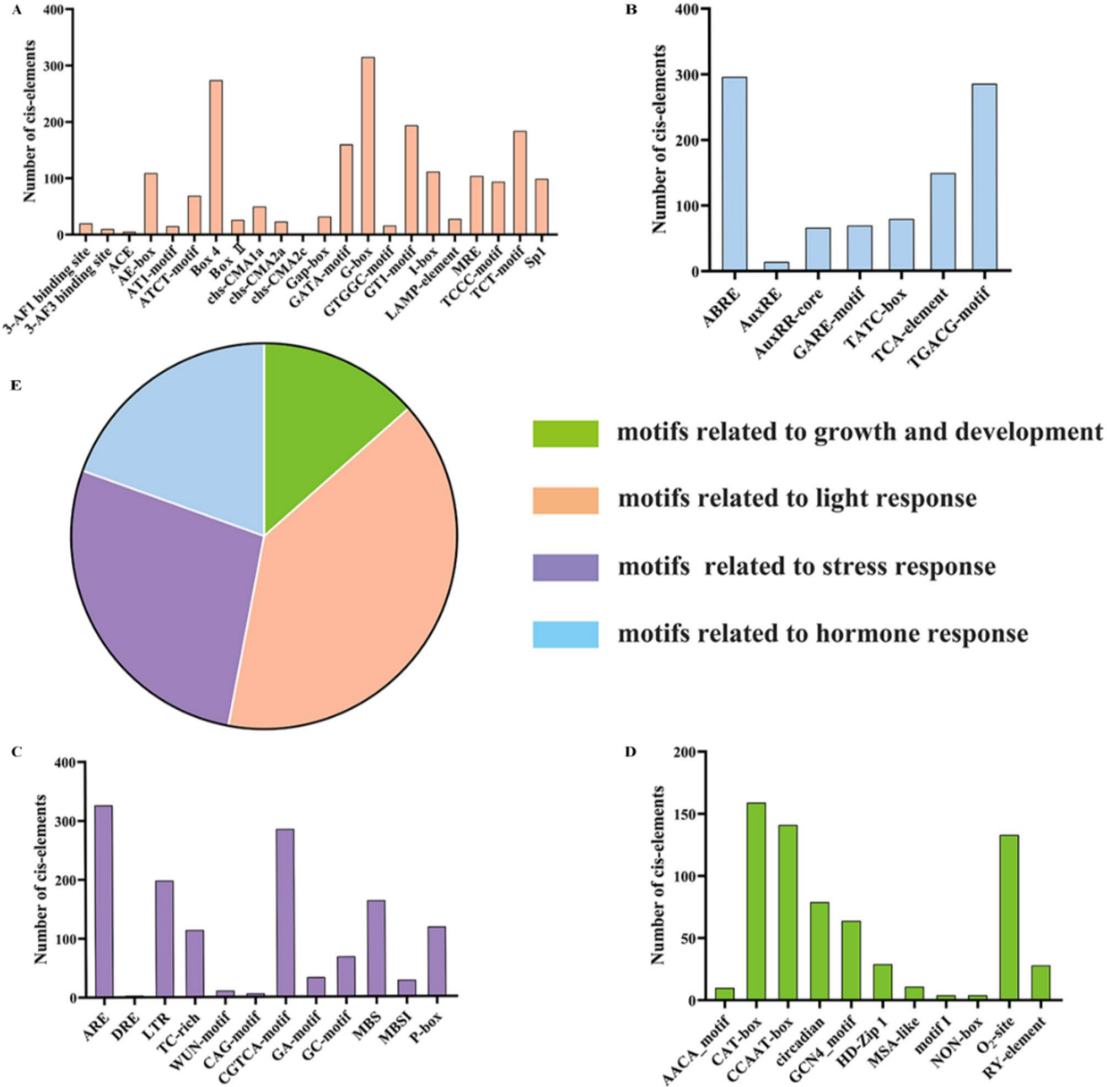
Frequency of *cis*-regulatory elements in the 2.0 kb upstream regions of VcUGTs. **A** *Cis*-regulatory elements related to light response. **B** *Cis*-regulatory elements related to hormone response. **C** *Cis*-regulatory elements related to stress response. **D** *Cis*-regulatory elements related to growth and development. **E **The categorized four types of *cis*-regulatory elements。

To analyze the *cis*-regulatory elements in *VcUGT* family genes, the 2000 bp upstream sequence of each *VcUGT* family genes was extracted and analyzed by the PlantCare online webserver (Table S2). The results indicated that the promoter regions of the blueberry *VcUGT* genes contain a total of 43 types of promoter elements, which can be classified into light-responsive elements, growth and development-related elements, hormone-responsive elements, and abiotic stress-related elements (Fig. 4). Light-responsive elements constitute the majority of *cis*-acting elements in the promoters of blueberry *VcUGTs*, including G-box and Box-4 elements, indicating that *VcUGT* genes may influence by light conditions. Additionally, the promoter regions contain regulatory elements associated with plant growth and development, such as NON-box and CAT-box elements related to tissue-specific expression, RY elements related to seed-specific regulation, GCN4 and AACA elements associated with endosperm expression, and circadian and MSA elements involved in circadian rhythm control and cell cycle regulation.

### Chromosome location and duplication of *VcUGT* family genes in blueberry

**Fig. 5:**
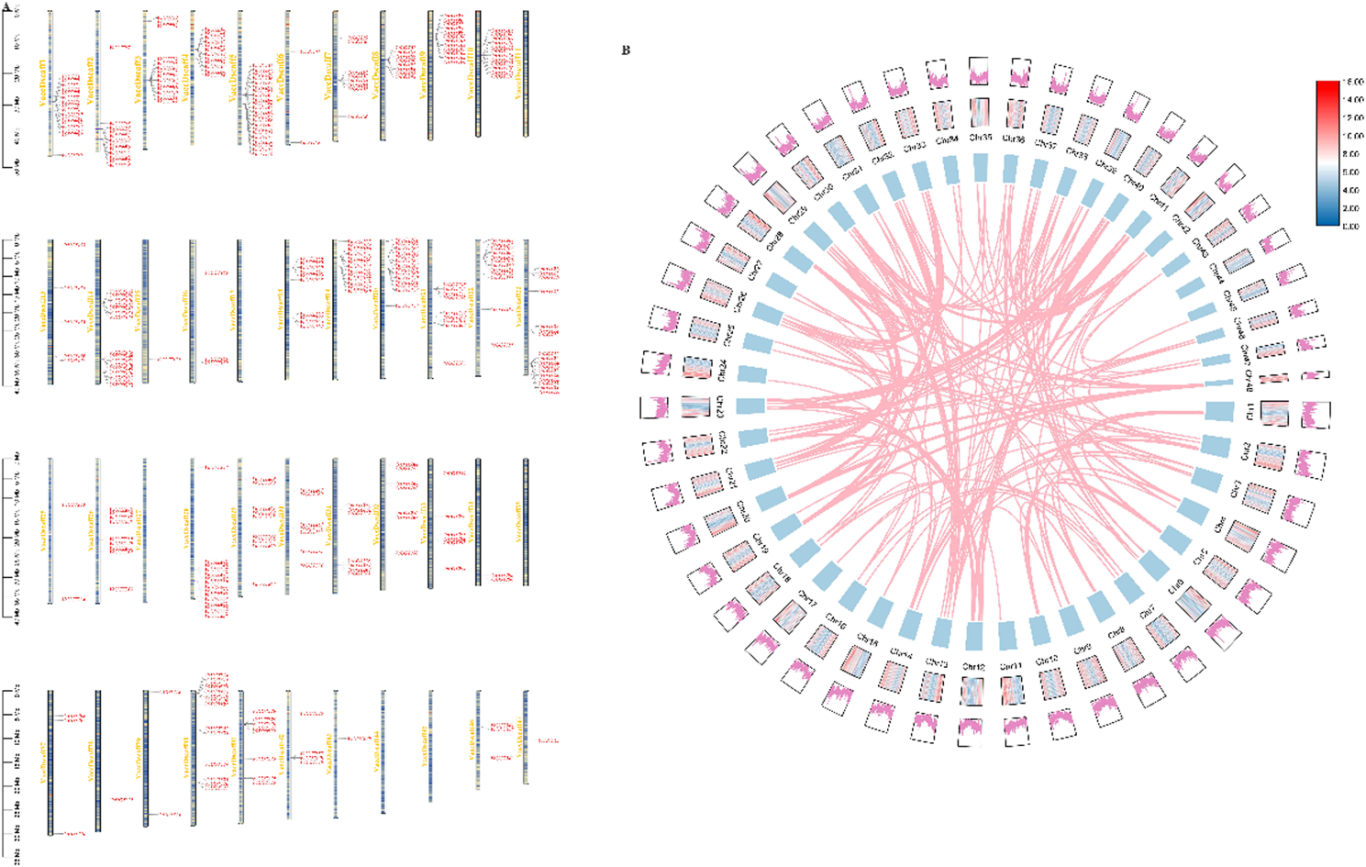
The distribution and collinearity analysis of the VcUGT genes on the chromosomes in blueberry. **A** The distribution of the VcUGT genes on the chromosomes in blueberry the genetic distance of seven chromosomes were represented by the scale in megabases (Mb) on the left. **B **Syntenic relationship of UGT duplicated gene pairs in blueberry. The boxes represent chromosomes and the lines represent UGT homologous pairs. The line map and heatmap of the outer rings represent the density of genes on chromosomes

In the course of evolution, plants commonly employ gene duplication as a means to expand gene content. To investigate their chromosomal distribution and evolutionary relationships, including detecting duplication events such as tandem duplication, segmental duplication, and whole genome duplication (WGD), we analyzed the positions of *VcUGT* genes on the 48 blueberry chromosomes. As shown in Fig. 5, the distribution of *VcUGT* genes across the 48 chromosomes of blueberry was uneven, with chromosome 5 showed the highest abundance of *VcUGT* genes (22 genes) and several chromosomes containing only one gene or none at all. Chromosomes 1, 2, 3, 4, 5, 8, 10, 14, 18, 22, 30, 36, and 48 contain densely distributed and evolutionarily conserved *VcUGT* genes, whereas a few genes showed dispersed distribution, particularly on chromosomes 13 and 22. To reveal the expansion mechanism of the *UGT* genes in the blueberry genome, gene duplication analysis was performed. About 313 genes involved in gene replication events, 286 in fragment repetition events, and 27 in tandem repeats. The results indicate that genome-wide and fragment replication played a pivotal role on the amplification of *VcUGT* family in evolution.

### Gene expression analysis by RT-qPCR data of *VcUGTs *during blueberry fruit development

**Fig. 6:**
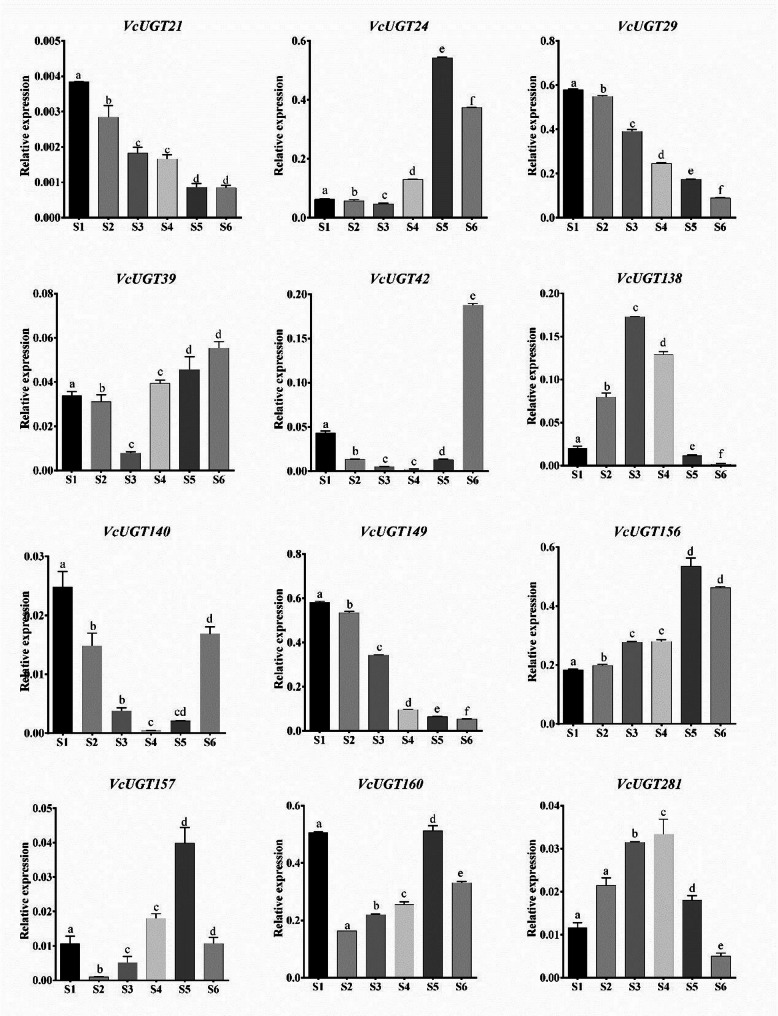
Expression analysis of 14 *VcUGT* genes from Group G during blueberry fruit development. Error bars represent the standard deviation *n* = 3. Different lowercase letters indicate significant differences at *P* < 0.05

Based on the published RNA-seq expression data (PRJNA494180), 14 *VcUGT* genes with relatively high expression levels were selected from the G group, and their expression patterns during different developmental stages of fruits were analyzed. As shown in Fig. 6, the expression patterns of *VcUGT* genes in various developmental stages of blueberry fruits differ. Among them, *VcUGT21*, *VcUGT29*, and *VcUGT149* showed a gradual decrease in expression levels during fruit development, with the highest expression levels observed in S1 stage. *VcUGT39*, *VcUGT42*, and *VcUGT140* exhibited a trend of initially decreasing and then increasing expression levels, though their overall expression levels were lower. *VcUGT24*, *VcUGT138*, *VcUGT156*, *VcUGT157*, *VcUGT174*, and *VcUGT281* showed a trend of initially increasing and then decreasing expression levels. *VcUGT24 and VcUGT156* exhibited relatively high expression levels throughout the entire fruit development process, indicating a low correlation with fruit development. Notably, *VcUGT160* showed higher expression levels in S1 and S5, suggesting its association with the early and mature stages of fruit development.

### Subcellular location of *VcUGT160*

**Fig. 7:**
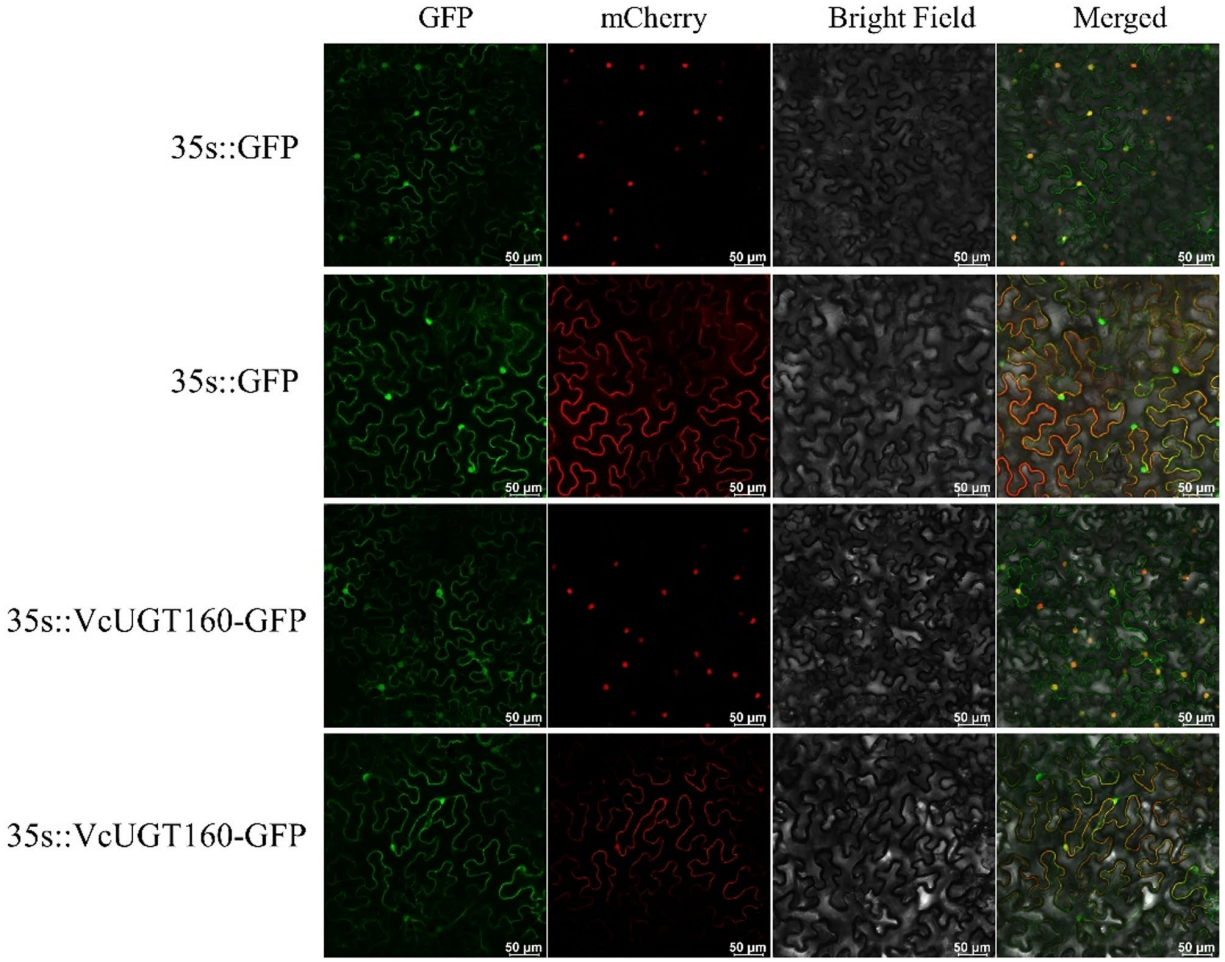
Subcellular localization of VcUGT160 using transient expression system in tobacco leaves. The VcUGT160-GFP (Green Fluorescent Protein) fusion construct was localized to the nucleus and plasma membrane. Scale bar = 50 mm

We selected the *VcUGT160*, which exhibited a specific expression pattern during fruit development, for further study. To determine its expression location, we initially used the PROTCOMP online tools for computational prediction. The results showed that VcUGT160 was localized in both the cytoplasm and the nucleus. Subsequently, the expression location of the VcUGT160-GFP fusion protein was verified using the tobacco transient transformation system. As shown in Fig. 7, the VcUGT160-GFP recombinant vector displayed prominent nuclear and plasma membrane fluorescence signals, suggesting that the protein encoded by *VcUGT160* is expressed in the nucleus and plasma membrane.

### Function analysis of the *VcUGT160* gene by VIGS assays

**Fig. 8:**
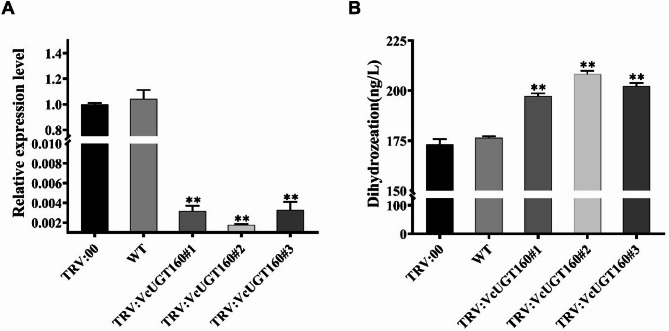
Virus-induced gene silencing of *VcUGT160* in blueberry. **A **The expression of VcUGT160-silenced blueberry fruits. pTRV2 was injected with Agrobacterium harboring an empty vector. Data are the mean ± SE (*n* = 3). **B** The relative dihydrozeatin content in fruits of VcUGT160-silenced blueberry fruits. pTRV2 was injected with Agrobacterium harboring an empty vector. Data are the mean ± SE (*n* = 5)

Virus-induced gene silencing (VIGS) is a powerful tool for the study of gene function. To further investigate the function of *UGT* gene in blueberry, gene silencing analysis of *VcUGT160* was performed by VIGS. In order to determine whether *VcUGT160* was effectively silenced, the total RNA was extracted from single fruit of blueberry after injection, and the expression of *VcUGT160* in VIGS line, control line and WT was detected by RT-qPCR. As shown in the Fig. 8A, the expression level of *VcUGT160* in blueberry fruits VIGS lines was significantly lower than the control line, but there was no significant difference between the WT and the control group.

According to evolutionary analysis and functional prediction, *VcUGT160* belonged to UGT85A2 family and mainly involved in the glycosylation of dihydrozeatin (Fig.S1). We used enzyme-linked immunosorbent assay to detect the content of dihydrozeatin in fruits. As shown in Fig. 8B, no significant difference was found between the wild type and the control group, which were 174.39 ng/mL and 176.41 ng/mL, respectively, while the dihydrozeatin content in the VIGS line was significantly higher than that in the control group. The dihydrozeatin contents of the three VIGS lines were 196.63 ng/mL, 208.99 ng/mL and 204.065 ng/mL, respectively. These results suggest that the silencing of *VcUGT160* lead to the change of dihydrozeatin content, and *VcUGT160* play an important role in the regulation of fruit development mediated by dihydrozeatin.

## Discussion

Plant UDP-glycosyltransferases (UGTs) have garnered considerable attention due to their vast numbers and diverse functions. The superfamily has been profiled in a variety of plant species, including model plants Arabidopsis (*Arabidopsis thaliana*) [28], rice (*Oryza sativa*) [29], as well as in fruit plants grapes (*Vitis vinifera*) [30], kiwifruit (*Actinidia chinensis*) [31], peach (*Prunus persica*) [32], and pear (*Pyrus communis*) [33].

In our study, 361 VcUGT members have been identified. The number of blueberry UGTs is greater than that in many other plants. The relative expansion of *VcUGT* gene family was related to the whole genome-wide duplication and segmental duplication. Phylogenetic analysis revealed that 361 VcUGTs clustered into 12 groups, with groups C, I, J, K, N, and Q absent. Similar patterns were observed in soybean [34] and Arabidopsis [28] These absences in certain taxa likely occurred during plant evolutionary. Groups A, L, G, and E were identified as the most rapidly evolving groups in higher plant evolution, comprising 238 members and accounting for 65.92% of the gene family. This finding aligns with previous research on apples [5], indicated the conservative domains and exon-intron structures result in alterations in gene function. *VcUGT* gene in blueberry demonstrates significant conservation within their clade, with most genes in the same group exhibiting identical or similar exon/intron structures. This observation aligns with findings in tomato (*Solanum lycopersicum*) [35] and petunia (*Petunia*) [36], indicating a high level of conservation among UGT members within the same group across different species. Moreover, our study identified 10 conserved motifs including a typical UGT PSPG-box domain (Motif 1). Research suggests that the residues of the PSPG motif could directly interact with sugar donors, influenced the activity and specificity of glycosylation reaction [37, 38]. The specificity of blueberry *VcUGT* genes to sugar donors and acceptors across different evolutionary clades may contribute to the high functional differentiation of *UGTs* in various groups. Furthermore, the promoter sequence upstream of the *VcUGT* gene contains various *cis*-acting elements, including light-responsive elements, plant hormone-responsive elements and stress-related *cis*-acting elements, consistent with findings in *Quercus spp.* [39], and *Dendrobium officinale* [40], suggesting that the expression of *VcUGT* genes may be induced by plant hormones and environmental factors.

Previous research has demonstrated that cytokinin, including zeatin, are crucial in promoting the enlargement of blueberry fruits [41]. Notably, within group G, the UGT85 family is associated with cytokinin regulation and zeatin metabolism [20]. Until now, only limited research on the UGT85 family, with a few studies indicating that the UGT85 family in Arabidopsis was associated with cytokinin [13], while in grape [42] and peach [43], was related to the volatile compound linalool. To investigate the potential relationship between blueberry fruit expansion and cytokinin glycosylation, we conducted a quantitative analysis of the *UGT85* family members in group G. Among the 14 *VcUGT85* family members, significant differences in expression were observed during blueberry fruit development, indicates genes in G group may have regulatory functions in various stages of fruit development. To further explore the roles of *VcUGT85* family genes, we selected a member exhibiting a unique expression pattern during both the early and mature stages of fruit development for detailed analysis.

The function of UGT family members is closely related to their location, previous study has shown that plant UGTs had different location due to their diverse roles in catalyzing glycosylation reactions. The cytoplasm is the most common functional location for UGTs in plants [42, 44, 45], although research also indicates functional roles of this protein family in the cell membrane, mitochondria, and chloroplasts [46]. Some UGT members exhibit multiple functional locations, PpUGT85A2 in peach was located in cytoplasm and nucleus [43], CsUGT78A15 in tea was located in the endoplasmic reticulum and nucleus [47], PbUGT72AJ2 in pear was located in the cell membrane and cytoplasm [48]. In our study, *VcUGT160* encoded protein was found to localize in the cell membrane and nucleus, suggesting potential regulatory functions in these cellular compartments. UGT85 family function primarily involved in the glycosylation of secoiridoid compounds and the modulation of trans-zeatin on plant growth and development. In petunias, PhUGT85A96 demonstrates strict substrate specificity for 2-phenylethanol and benzyl alcohol [48]. UGT85K11, isolated from tea leaves, exhibits high catalytic activity in the glycosylation of eugenol, benzyl alcohol, and geraniol [49]. In *UGT85A1* overexpression Arabidopsis lines, the accumulation of trans-zeatin O-glucoside is significantly increased, potentially affecting the homeostasis of trans-zeatin [50]. Based on phylogenetic clustering and KEGG pathway prediction (Fig S1), we silenced the *VcUGT160* gene using the TRV virus and detected the content of dihydrozeatin in blueberry fruits. The results showed a significant increase in dihydrozeatin content, consistent with reports of altered trans-zeatin metabolism due to overexpression of *UGT85A1* in Arabidopsis, suggested that the *VcUGT160* gene could play an important regulatory role in dihydrozeatin glycosylation during fruit development, potentially influencing blueberry fruit development.

## Conclusion

In this study, a total of 361 *VcUGT* genes were identified, which were divided into 11 groups according to the classification of other plants. The chromosomal distribution, conserved motifs, exon/intron location and *cis*-elements analysis of the *UGT* gene family provide a useful material (structural) basis for understanding the function of the UGT gene family. According to the expression profile, 14 *VcUGT* genes with relatively high expression levels were selected from the G group as candidate genes, and *VcUGT160*, which is highly expressed during both the early and late stages of fruit development, was chosen as the target gene for further validation. Subcellular location and VIGS demonstrated that *VcUGT160* function in the cell membrane and nucleus, where it regulates the glycosylation of dihydrozeatin.

## Materials and methods

### Plant materials

The different fruit development stages S1 (fruit development stages S1), S2 (30 days after anthesis), S3 (ginkgo), S4 (powder fruit), S5 (blue fruit) and S6 (purple fruit) of blueberry variety “Northland” were collected in the small berry germplasm resource garden of Jilin Agricultural University. All plant materials were snap-frozen in liquid nitrogen and stored at −80 °C until used.

### Identification of *UGT *family genes

The Hidden Markov Model (HMM, PF00201) profile of the UGT family was downloaded from the Pfam database (http://pfam.xfam.org/) and used to identify UGT proteins in Blueberry genome using simple HMM search program in TBtools. The candidates were manually identified based on the information from the NCBI Conserved Domain database (http://www.ncbi.nlm.nih.gov/cdd/) and SMART database (http://smart.emblheidelberg.de/.server)(http://www.ncbi.nlm.nih.gov/Structure/cdd/wrpsb.cgi). Finally, members lacking the typical UGT structural domain were removed.

### Phylogenetic analysis

The UGT family sequence of Arabidopsis was obtained from TAIR database and other fruits plant sequence were obtained from NCBI database. Multiple sequence alignment of UGTs protein sequences was conducted by Muscle software, and the phylogenetic tree was constructed based on the alignment of the PSPG domains using TBtools and maximum likelihood (ML)methods with 1000 bootstrap [51]. Tree files were viewed and edited using ITOL online tools(https://itol.embl.de/).

### Conserved motif and gene structure analysis of VcUGTs

Full-length protein sequences of candidate VcUGTs were analyzed using MEME software (http://meme-suite.org/tools/meme) with the following parameters: the maximum value of motifs was set to identify 10 motifs, the minimum motif width was 6, and the maximum motif width was 200, and the obtained conserved motifs were annotated by SMART. Intron-exon map of VcUGTs was constructed according to genome annotation file and all of the results were visualized by TBtools [51].

### *Cis*-regulatory element analysis of promoters

The 2000-bp upstream sequence from the translation initiation codon of each *VcUGT* genes was obtained from the genomic data of blueberry were selected as the promoter regions and submitted to PlantCARE (http://bioinformatics.psb.ugent.be/webtools/plantcare/html/) to identify the *cis*-acting elements and then visualized after statistics.

### RNA isolation and quantitative real-time PCR analysis

The expression levels of 12 genes in different fruit development stages were analyzed using RT-qPCR. Total RNA was extracted using modified CTAB method [52], and its purity and concentration were evaluated by the Implen P330 (IMPLEN, Munich, Germany) instrument. Then the first-strand cDNA was synthesized using Takara PrimeScript™ RT reagent Kit with gDNA Eraser(For Perfect Real Time)(Takara, Dalian, China), and preserved at −20 °C. A set of gene primers for the blueberry *VcUGTs* genes was synthesized based on the information obtained from the QuantPrime [53] online tools and each primer pair was generated away from the conserved domain of the genes. The internal reference genes of blueberry used in the RT-qPCR analysis included the *Ef1α* gene [54]. All RT-qPCR primers were synthesized by Genewiz Bio-Technology Services Co. (Suzhou, China) (Table S3). RT-qPCR was performed with TB Green™ Premix Ex Taq ™ II (TaKaRa, Dalian, China) using the ABI Step One Plus real-time PCR machine. Following the manufacturer’s instructions, a 20 µL reaction mixture was prepared with 2.0 µL cDNA template, 0.4 µM primers (F/R), 0.4 µL ROX dye(100x), 10 µL master mix, and RNase-free water. PCR conditions were set according to the manufacturer’s protocol. Each experiment was repeated for biological replicates of the fruit’s samples. The relative transcript abundance values were calculated using the 2^−ΔCt^ method [55].

### ORF cloning and subcellular localization

The open reading frame of the *VcUGT160* (without a stop codon) (Table S3) was amplified from cDNA and cloned into pGDG-GFP by using pEASY^®^-Basic Seamless Cloning and Assembly Kit (TransGen Biotech, Beijing, China). The empty vector, recombinant plasmids and nuclear localization marker were transformed into Agrobacterium GV3101(pSoup-p19) competent cells by the freeze-thaw method, respectively. Subcellular localization was performed by transient expression of the VcUGT160-GFP then infiltrated into leaves of tobacco plants, as described previously [56]. After being cultured for 48 h under long-day conditions of 16 h light, the cells were observed with a laser con-focal microscope (TCS SP8, Leica, Germany).

### Construction of the VIGS lines

To silence *VcUGT160* gene in fruit of blueberry the VIGS vector was constructed. A specific cDNA fragment of *VcUGT160* were cloned into the *Eco*R I/*Bam*H I-digested pTRV2 vector. Then, the vector plasmid of pTRV1, pTRV2, and pTRV2-VcUGT160 were transferred into *Agrobacterium* strain (EHA105). The mixture solution of pTRV1 and pTRV2-*VcUGT160* was used to infect *‘Northland’* blueberry fruits. Meanwhile, the mixture solution of pTRV1 and pTRV2 was used as control vector. The fruit samples surrounding the injection site were collected after 20 days.

### Silencing efficiency analysis and dihydrozeatin content determination

The expression level of *VcUGT160* in the silenced lines was detected by real-time RT-qPCR, and the down-regulated plants were selected for subsequent observation and research. Successfully silenced fruits and control fruits were selected for measuring dihydrozeatin content. The dihydrozeatin content was determined using the protocol outlined in the dihydrozeatin ELISA detection kit manual.

### Statistical analysis

Relative gene transcript levels were analyzed using IBM SPSS statistics version 26.0 (IBM Corp., Armonk, NY, USA). Significant differences among means were determined using Duncan’s multiple range test at a significance level of *P* < 0.05. Differences in silencing efficiency of VIGS lines and dihydrozeatin content were analyzed by analysis of variance (ANOVA) using GraphPad Prism 6.0 (GraphPad Software Inc., LLC, 225 Franklin Street, USA).

## Supplementary Information

Supplementary Material 1.

## Data Availability

The datasets generated and/or analyzed during the current study are available in the Genome Database for Vaccinium, https://www.vaccinium.org/.
