# Supplementary figures and images for: Genome-wide characterization of the UDP-glycosyltransferases (UGT) family and functional analysis of VcUGT160 involved in dihydrozeatin glycosylation during blueberry fruits development
Source: BMC Genomics. 2025 Nov 14;26:1044. doi: 10.1186/s12864-025-12267-5 (PMC12619228; doi:10.1186/s12864-025-12267-5)

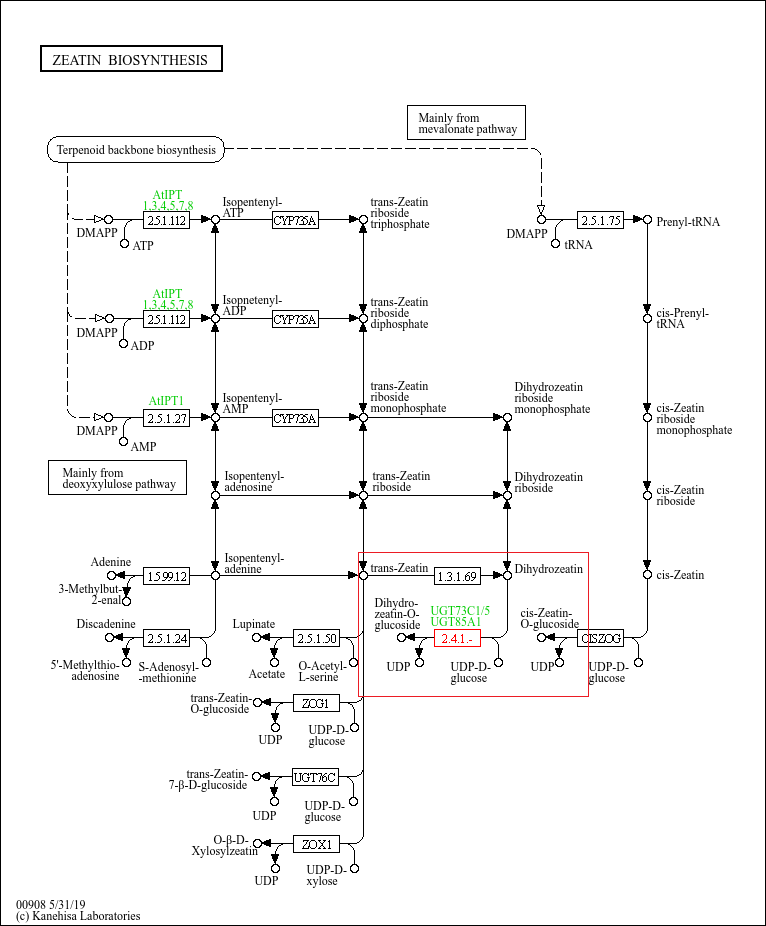


Supplementary Fig. 1 Prediction diagram of dihydrozeatin glycosylation pathway

Supplement: Supplementary file 1 — Supplementary Material 1. [file 12864_2025_12267_MOESM1_ESM.zip › Supplementary material/Supplementary Figures.docx]
